# An International Review of Workplace Violence Against Healthcare Providers: Sudan as a Case Study

**DOI:** 10.7759/cureus.52355

**Published:** 2024-01-16

**Authors:** Salah Eldin A Abdel Haleem, Haitham M El Bingawi

**Affiliations:** 1 Department of Pharmacology and Therapeutics, University of Bahri, Khartoum, SDN; 2 Department of Pharmacology, Al-Baha University, Al-Baha, SAU; 3 Department of Medicine, Sultan Qaboos University, Muscat, OMN

**Keywords:** workplace violence, khartoum, healthcare facilities, healthcare providers, sudan

## Abstract

Workplace violence experienced by healthcare providers (HCPs) in Sudan has gone viral, driving many HCPs outside the country. Globally, HCPs have accepted workplace violence as a phenomenon integral to their clinical work, causing an underreporting of incidents. This study reviews the causes and explores solutions for the phenomenon. Search strategies were conducted using internet sources including PubMed, Embase, Google Scholar, and Cochrane. References to Sudan were limited to HCPs in public healthcare facilities. A descriptive analysis was conducted on the global status of workplace violence toward HCPs. Future interventions were examined and discussed considering Sudan’s circumstances. Results showed the "pandemic" nature of the phenomenon. Workplace violence contributes to the deterioration of the quality and efficiency of the healthcare system with consequences for effective healthcare delivery. It is concluded that a multiapproach intervention needs to be integrated to circumvent the standing multifactorial situation. Statutory actions are needed towards the widespread violence and impunity. Workplace organizational procedures are needed to address the patient’s needs that overwhelm scarce resources. Robust educational efforts are recommended by HCP training bodies, the media, and other stakeholders to improve the doctor/patient relationship.

## Introduction and background

On January 27, 2017, a Sudanese specialist in dermatology and venereal disease was stabbed to death by one of his patients in his clinic in Sinnar, 240 kilometers south of Khartoum. The incident shocked the specialist’s colleagues and the local community in which he had been a respected doctor with 20 years of service in state public hospitals. Although this was the first loss of life to workplace violence against a healthcare provider (HCP), workplace violence toward HCPs has become an escalating problem in a country devastated by instability and war. Previous investigators noticed that violence in the healthcare system can mirror violence in society in general [1,2]. Poverty, instability, and lack of opportunities to generate income have caused millions to move to large cities where they join the unemployed population that includes millions of high school and university graduates. Khartoum now consists of a young, mostly male, community who have lost the tolerance, empathy, and sympathy that has long characterized its people. The neglected public sector has been overwhelmed, either by the decay of its infrastructure, or the failure of its establishment to accommodate change, qualitatively and quantitively. Public healthcare institutions are no exception. They are busy, crowded, and noisy with decreased capacity and a reduced ability to serve the public competently. Most reporters have classified workplace violence into verbal and physical abuse, threats, and sexual harassment [1,3]. Others have described verbal abuse as emotional attacks that include verbal abuse, in the form of harsh words, cursing, speaking aggressively, or a raised voice [4]. For many reasons, it is difficult to estimate the countless incidents of violence in Sudan using these classifications. While Sudanese HCPs think of violence toward HCPs as a local phenomenon, data from the European Agency for Safety and Health at Work indicate that healthcare is the sector that experiences the highest rates of workplace violence [4]. It is a common local and global belief [2,5,6] that violence and aggression come with the job. This causes under-reporting of these incidents in addition to lack of support from seniors, and unclear reporting procedures and institutional policies [5]. This work was undertaken to highlight the phenomenon of workplace violence toward HCPs in Sudan, to understand its underlying causes, and to find possible solutions.

Methods

Search strategies were conducted using various internet sources including PubMed, Embase, Google Scholar, and Cochrane, and the results were proactively reviewed. The international review engaged peer-reviewed reports of aggression toward HCPs. They were sub-grouped according to whether they describe aggression and its impact on healthcare systems, investigate the causes of aggression, or suggest solutions. The Sudan’s review included reports of aggression/violence toward HCPs. They were sub-grouped according to whether they describe aggression/violence and its impact on the healthcare system, investigate the causes of aggression/violence, or suggest solutions. The Sudan's review included reports of aggression/violence toward HCPs. Single case reports or opinion publications were excluded. Foreground questions were formulated to retrieve information. Search terms like verbal, physical, and psychological aggression, or violence from a patient or co-patients (patient companions) were used to define the act against the HCP. Answers were sought for questions that had included the following descriptors and Boolean operators: Definition (and/or) legal definition of workplace violence (and/or) aggression against doctors (and/or) healthcare providers; acts (and/or) incidents of violence (and/or) aggression against doctors (and/or) healthcare providers; reasons for (and/or) causes of violence (and/or) aggression against doctors (and/or) healthcare providers; outcomes of violence (and/or) aggression against doctors (and/or) healthcare providers; solutions to (and/or) measures taken against workplace violence against doctors (and/or) healthcare providers. The same descriptors and Boolean operators were used in the section about Sudan with the addition of the term "in Sudan." No specific timeframe was added to the search questions. The target population was defined as HCPs in any healthcare facility. However, for Sudan, we referred only to HCPs in public healthcare facilities. Keywords like physician, doctor, healthcare provider, and general practitioner were used to define the target population. The authors collaborated on screening all retrieved reports. Data were categorized according to the four areas that answered the research questions. No specific automation tools were used in the process. During data collection, as has been anticipated by the authors, few peer-reviewed reports were retrieved from the primary, secondary, and tertiary sources of literature pertaining to aggression against HCPs in Sudan. Thereafter, criteria for report inclusion in the section of the review on Sudan have been widened, sparingly and carefully, to include statistical data from government websites and website material from professional associations and press releases in the local newspapers. A descriptive analysis was conducted on the local and international status of incidents of aggression/violence toward HCPs, causes common to these incidents, and steps taken toward solutions.

## Review

International review results

Definitions

The World Health Organization (WHO) defines violence as “the intentional use of threatened or actual force against a person or a group which may cause physical or psychological trauma.” The European Commission defines workplace violence as “all situations when a worker is offended, threatened, or attacked in conditions directly related to his/her job and when these situations directly or indirectly endanger his/her safety or involve an explicit or implicit challenge to his/her well-being or health” [4]. The International Labour Organization (ILO) uses a wider definition: “incidents where staff is abused, threatened or assaulted in circumstances related to their work” [4]. The British Health and Safety Executive defines violence as “any incident in which an employee is threatened or assaulted by a member of the public in circumstances arising out of the course of his/her employment” [7]. The Norwegian Labour Inspection Authority defines it as “incidents where an employee is abused, sexually harassed, or assaulted in circumstances relating to their work, involving an explicit or implicit challenge to their safety, well-being or health” [3]. It has been reported that violence is physical assault with intent to harm, not all aggression leads to violence, and violence is a step further than aggression [6].

Incidence and Characteristics of Workplace Aggression Against HCPs

In the UK, there are reports from 1994 of a fatal stabbing at work of a Scottish general practitioner [2]. Furthermore, it is estimated that 5% of National Health Service (NHS) HCPs have been threatened with a weapon. Despite the risks and legal obligations, only 43% of hospitals have a policy on violence, 3% offer special staff training, 50% provide no training, 25% advise staff on reporting procedures, and 87% of health service staff are worried about workplace violence [2]. A British Medical Association survey estimated that 50% of obstetricians and gynecologists had experienced violence in the preceding year where the perpetrator was the patient (50%), a relative (34%), or a friend (14%) [8]. Estimates of workplace aggression toward HCPs amounted to 81% for Ireland [3], and 82% for Spain [3]. The National Report on Aggression to Physicians in Spain 2010-2015 estimated that there were 2419 incidents of aggression toward physicians, 51% toward men, 54% in primary care departments, 89% in the public sector, 33.3% toward professionals aged 46-55, 70% perpetrated by the patient, and 30% perpetrated by a relative or companion [9]. In Germany, one report estimates incidents at 81.4% and 70.7% for verbal or physical aggression [10]; while another reports them at 91% [4]. A German study reports that 91% of primary care physicians have been subjected to aggression, 23% were objects of severe aggression or violence, and the annual prevalence of aggression and violence against primary care physicians is lower than against police officers (54% versus 80%), but much higher than against teachers (47% versus 2%) [10]. Of about 10,000 Australian clinicians, 70.6% reported experiencing aggression, 32.3% of which had been acts of physical aggression [11]. A 12-month Belgian survey of 3726 HCPs reported that 37% were victims of aggression, 33% verbal, 30% psychological, 14% physical, and 10% sexual. Women, younger physicians, and psychiatric and emergency departments were more commonly the subjects of aggression. On December 1, 2015, a 64-year-old family physician was murdered during a house call to a patient [6]. A Norwegian primary care center study involving 536 HCPs estimates that 78% of them had been verbally abused, 44% had been exposed to threats, 13% had been physically abused, and 9% had been sexually harassed for 12 months. Significantly more nurses were associated with verbal abuse, and males were at a higher risk for physical abuse [3]. A Polish study estimated that 12% of junior doctors, 9% of seniors, and 2% of managers reported physical assault [12]. In Austria, 7.8% of hospital workers were subjected to terror and abuse in the workplace, and 36- to 45-year-old employees at teaching hospitals and specialists experienced physical violence [13,14]. An Italian study reports the magnitude of aggression at 33.3% and physical assault at 10% [4]. In Poland, patients’ aggression was inflicted on 92% of nurses, 86% of doctors, and 74% of midwives [4]. In Istanbul, 66.8% of the respondent HCPs reported that they had been exposed to violence and aggression during the last year, 73.1% had witnessed aggression, less exposure to aggression was detected among the 40-49 age group, and 60.3% had suffered from aggression committed by patient’s relatives [15]. A large study among Canadian physicians showed that 98% had experienced minor aggression, 75% severe aggression, and 39% very severe aggression [6]. Nationwide American research determined that 75% of physicians in emergency departments had experienced violence [6]. A Michigan study estimated physical assault at 11.7% of HCPs, particularly female doctors [3]. Between 1980 and 1990, over 100 healthcare workers died because of violence [10] and in 170 university hospitals, 57% of all emergency room employees had been threatened with a weapon over five years [16]. In California, non-patients were responsible for nearly one-quarter of all violent episodes [17]. In China, violence against physicians has witnessed a surge [6]. A 2019 survey showed that 85% of HCPs were subject to aggression and 13% had been physically assaulted [9]. In June 2010, a Chinese doctor and nurse were fatally stabbed in Shandong province by the son of a patient who died of liver cancer 13 years prior, and a pediatrician in Fujian province was injured after leaping out of a fifth-floor window to escape angry relatives of a newborn baby who had died under his care [10]. A female doctor in Tuticorin, India, was killed by the husband of a pregnant woman who was admitted in a serious condition. The husband entered the doctor’s consultation room with three accomplices and attacked her with a sword [10]. Violent incidents in Japan are estimated at 0.20×10^-3^ events per practice hour [4]. In Iraq, 80% of HCPs reported an assault by a patient or family member, 38% of which involved a gun, 87% were resident physicians, and 86% reported that it was emotionally painful to talk about their experience [18,19]. A Pakistani study found that 73.8% of HCPs (n=250) were victims of violence in a 12-month period and that 71% of the assailants were patients’ relatives [4]. A systematic review concluded that the most common type of workplace violence is where the aggressor is a patient or a relative of the patient. These events are categorized in the literature as "type II workplace violence" [20].

Causes of Workplace Aggression Against HCPs

One report of over 4,000 HCPs attributed 39% of incidents to staff behavior, 26% to patient/visitor behavior, 17% to organizational conditions, and 10% to long waiting times [1]. The bombings at a Massachusetts Planned Parenthood clinic and the Alfred E. Murrah Federal Building in Oklahoma City [7,21] suggest that HCPs can be targets for politically motivated acts of violence or terrorism. It has been proposed that aggression toward HCPs increases parallel to the increased prevalence of violence in society [2]. In India, hospital violence toward workers and property is often used by local politicians as a show of strength, and to avenge the death of a loved one [10,22]. The global negative image generated by the media has contributed to the loss of traditional confidence in HCPs. This loss of respect for doctors has caused an increase in violent attitudes [9]. An Indian study reported that the image of doctors was harmed by impressions of profit-making on the part of doctors [23]. Additional contributing factors include an unrealistic expectation that paying more money should save one’s life, sensational news reports of doctors overcharging for various tests and reports, doctors being physically assaulted daily, and perpetrators not being punished [10,22]. Similar observations, including a lack of security, are reported in Pakistan [4]. HCPs themselves contribute to increased incidents of aggression in healthcare settings through a lack of skills in the prevention and management of aggression and violence [24], commonly associated with a shorter time in professional practice [6] and shift work intolerance syndrome due to overwork, emotional exhaustion, stress, and improper relations in the therapeutic team [12]. This finding is supported by the observation that physicians working in solo practice encounter less aggression compared to those working in groups [6]. Aspects related to therapeutic decisions, including report issuing, have been estimated to account for almost 25% of reported aggressions [10]. Patients or co-patients commit healthcare-related aggression in reaction to variable stimuli including drug intoxication/influence, mental illness, dissatisfaction with service (25%), pain, and anxiety [3]. This also applies to the USA [3], Australia [3], Spain [2], and Germany [3,10]. Patients’ and families’ stress caused by illness is another stimulus [4,25]. In addition, lengthy waiting times, frustrated co-patients, and the hours between 18:00 and 07:00 are considered the worst for the occurrence of aggression toward HCPs [2]. In India, financial issues were reported as reasons for patient or co-patient aggression, including unnecessary investigations, requests for advance payments, or withholding of deceased bodies until bill settlement [26]. Medical causes for patient aggression include hypoxia, delirium, adverse drug reaction, head injury, infection, cerebral irritation or hypoglycemia, withdrawal from alcohol and drugs, and pain [27]. Distress or unmet needs are other triggers for patient aggression [28], precipitated by unpleasant symptoms, sleep deprivation, communication problems, visual or hearing impairment, needing the toilet, cognition problems, restricted movement, and minimal access to fresh air or cigarettes [28]. A Japanese study attributed patient aggression to post-traumatic stress disorder [4]. Australian research suggests that cultural and communication issues may serve as triggers for aggression [11]. Considering patients, anxiety generates hyper-vigilance, inducing selective attentional bias for threats. It drives patients to make negative attributions regarding staff actions. Thus, it will invoke aggressive responses. What HCPs perceive as aggression may be seen by them as a defense against perceived attack [29]. On the other hand, institutional issues include unrestricted movement of visitors in hospitals [24], staff shortages, stress-tiredness [10,12,22], hospital location, gangs, easy access to triage, and perpetrator’s negative characteristics [30]. In China, violence has been attributed to the overall organization of the healthcare system [6]. In India, where violence associated with organizational aspects accounts for 15.4% of attacks, it has been blamed on the scarcity of resource allocation to the public sector (2% of the total budget) [9,10,22]. Miscellaneous attributes for healthcare-associated violence include ethnic discordance between patient and physician [31]. Gender, ethnicity, education level, and employment status have been excluded as predictors of violence [32,33]. However, a Polish study reports that 57% of HCPs could not identify a specific reason for aggression in their workplace [12].

Consequences of Workplace Aggression Against HCPs

Exposure to workplace aggression/violence impacts the careers of HCPs, as well as their ability to make decisions [11,26] and carry out day-to-day duties [23]. It also reduces their commitment to good care practice and undermines their confidence in their professional capabilities [34], increasing the probability of medical errors [6] and even malpractice [35]. Workplace violent incidents contribute to the deterioration of the quality and efficiency of the entire healthcare system [23] and generate consequences for patient care and effective healthcare delivery [25]. Additionally, exposure to workplace aggression leads to deterioration of the working environment with dissatisfaction and low productivity [36]. Doctors’ immigration to countries with allegedly safe work environments affects countries where violence predominates. Some studies reported a 22% decrease in the number of medical specialists in Baghdad between 2004 and 2007 due in part to violent deaths, threats, and kidnappings [18,19]. In addition to these effects on the healthcare system, workplace aggression impacts the personal well-being of HCPs causing depression, insomnia, posttraumatic stress, fear, and anxiety, which lead to absenteeism [10]. Many have lost their clinics, injured themselves, lost lives, and tarnished their reputations due to these incidents [22]. In Spain, 20% of assaults were associated with personal injuries, up to 12% of attacks resulted in sabbatical leave, and 10% of cases, material damage occurred [9].

Sudan's review

The Study Context

The Republic of Sudan is an African country with a unique social structure consisting of different cultures, races, and accents. Khartoum is the capital, it consists of three cities, Khartoum (the political capital), Omdurman (the national capital), and Khartoum Bahri (the industrial capital). The city is divided and connected, simultaneously, by the Nile River, therefore, Khartoum is also called "the land of the two Niles" where the Blue Nile meets the White Nile in a unique scene. This unique location makes Khartoum one of the most geographically distinguished cities in the world. It was one of the most urbanized African cities in the 1960s [37,38], but due to the failure of successive governments to create balanced development in the countryside, Khartoum's circumstances have changed from being an urban city to a large village which is currently unable to provide basic life requirements for many of those seeking education, healthcare, and job opportunities. Additionally, the wars that broke out in some areas of Sudan led to waves of displacement toward Khartoum, increasing pressure on the city, which was unprepared to accommodate the arrivals [39]. Since early in this century, Sudan has suffered relentless political and economic isolation which has affected the lives and livelihoods of its people and has led to a vicious chain of failures in all aspects. Economic development has been disrupted, the standard of education and health services has declined, and young people have become frustrated and disappointed by growing unemployment affecting most sectors. The negative impact affected not only the economy, political situation, and infrastructure, but society has witnessed a change in the behavior and nature of the easygoing Sudanese personality. The new society has a greater tendency toward violence and rejection of one another. Furthermore, the evolution of tribalism is a reverse of the natural development of societies [40].

Health Services in Sudan

Sudan had one of the most efficient health systems compared to neighboring countries [41]. However, like other sectors, it has been in steady decline since the middle of the last century. The overall policy of enabling military security at the expense of supporting other services has resulted in a further deterioration of health services. Quality is important in providing a standard of care health service [42]. In Sudan, the working environment is suboptimal in most governmental hospitals and lacks the infrastructure to meet patient’s requirements as well as the organizational resources to support HCPs to work effectively and productively. HCPs often bear the burden of facing this fundamental defect in the system alone, and as a result, they become victims, together with their patients. Emergency departments are the most common site for violence against healthcare professionals in Sudan [43], most likely due to the poor working environment. The inappropriately equipped work environment has led to the continuous loss of highly qualified medical staff either to the private sector or to immigration [44]. Interns and registrars in training were left with no option but to work under these difficult circumstances. Due to the reduced doctor-patient ratio, they work far beyond their official working hours. According to the Sudan Medical Specialization Board, approximately 80% of registrars pay for their four-year specialization. If the Ministry of Health paid for their behavior, they must serve for eight years to receive their certificates [45]. These factors have led to disruptions in doctor training, not only from a clinical aspect but also other core aspects of the medical profession like ethics and communication skills. A question arises, if institutions are not obligated to pay salaries, are physicians obligated to work according to standard contracts? This dysfunctional situation was obvious during the COVID-19 pandemic as considerable numbers of doctors did not work. Despite the many possible reasons for their absence, one may be the feeling of not having a firm administrative obligation. A field study conducted in the major teaching hospitals in the state of Khartoum measured the quality of health services in government hospitals from the patients’ perspectives [46]. The results of this study, outlined below, provide insight into some of the reasons for violence against doctors: The first reason is that most patients would prefer to receive their medical care in private hospitals, but because of financial constraints, they have no choice but to attend government hospitals. The second reason is that government hospitals are not adequately equipped to provide health services. The third reason is that patients feel unsure of timely access to health services in government hospitals. The fourth reason is that patients fear that medical staff will not have the necessary medical competence to treat their diseases in government hospitals. The media is another issue, it plays a profound role in shaping public attitudes and opinions toward HCPs. On many occasions, the media choose marketing titles that tend to criminalize indirectly, or sometimes explicitly, the healthcare professionals or the facility before responsibly verifying the events. For example, a newspaper reported that a patient was hospitalized with tooth decay and died the next day [47]. Such headlines, unfortunately, shake the community’s confidence in the health sector, increase anger, and more importantly, create an assumption that every doctor is guilty until proven otherwise.

The Magnitude of the Problem

According to a report issued by the Central Sudan Doctors Committee, more than 50 cases of violence against HCPs in Sudan were observed from August 2019 to May 2020. We refer below to some of the attacks that occurred during this period, reflecting the magnitude of the problem faced by HCPs [43]. On August 18, 2019, a group of obstetrics and gynecology doctors at the Turkish Teaching Hospital in Khartoum were surrounded for six hours and beaten by co-patients. This event resulted in the complete withdrawal of the doctors. This was the eighth case of its kind in that hospital [43]. Parallelly, over five different attacks took place in a three-month period by co-patients against medical staff in the surgical department at Kassala Hospital [43]. On October 9, 2019, an assault against an obstetrics and gynecology registrar by a co-patient took place at Al-Nahoud Hospital [43]. Five assaults took place during two weeks in the emergency department of Wad Medani Teaching Hospital, the last of which led to the suspension of the emergency department on November 9, 2019 [43]. On November 10, 2019, an individual with a military status assaulted an emergency department doctor at Port Sudan Teaching Hospital [43]. On November 16, 2019, a physician at Al-Obayid Hospital was seriously injured by an individual with military status, leading to a general three-day strike by all the hospital’s doctors [43]. On November 16, 2019, a physician was assaulted by a co-patient at El-Fasher Hospital [43]. On December 1, 2019, an individual with a military status assaulted an administrative staff member in Bashaer Hospital [43]. On March 2, 2020, a doctor was assaulted by an individual with a military status at Muhammad al-Amin Hamid Pediatrics Hospital [43]. On March 3, 2020, an emergency surgeon at Al-Damazin Hospital was arrested while performing his work because he refused to obey the command of an individual with a military status. On March 20, 2020, verbal and physical assault targeted a physician at El-Fasher Specialist Hospital for Obstetrics and Gynecology, this led to a complete cessation of work in all state hospitals for the next 48 hours [43]. On March 25, 2020, the severe beating of doctors at the emergency department of Jabal Awlia Hospital resulted in a transient loss of consciousness of one of the doctors. This event resulted in the closure of all hospital departments and a general strike [43]. On April 1, 2020, a doctor was arrested and imprisoned when he violated a curfew in Khartoum State [43]. On April 4, 2020, a general practitioner was attacked by an individual with a military status at Ali Abdel Fattah Hospital, as no legal action was taken against the aggressor, all house officers withdrew from the hospital [43]. On April 11, 2020, a pediatric surgeon at Wad Medani Teaching Hospital was beaten by an individual with a military status [43]. On April 19, 2020, a physician in the Al-Hasahisa Hospital surgical department was attacked by co-patients because she asked them to leave the hospital due to a suspected case of COVID-19 [43]. On April 20, 2020, a doctor working in Kutum Hospital was sentenced and sent to prison over the criminal charge of unintentional killing instead of following the law and filing a complaint to the Sudan Medical Council [43]. On April 29, 2020, a surgical registrar was severely beaten by a co-patient at Omdurman Teaching Hospital [43]. On May 21, 2020, an emergency department physician at the East Nile Hospital was attacked by co-patients, resulting in the temporary discontinuation of hospital service [43]. On May 21, 2020, a physician was assaulted by an individual with a military status at Bahri Teaching Hospital [43]. On May 21, 2020, a group of co-patients attacked the Omdurman Teaching Hospital emergency room, resulting in physical damage to hospital property. The policeman on guard was also injured [43]. In response to deteriorating living conditions and defective policies, citizens organized massive protests in December 2018. The government confronted the protests with excessive violence including HCPs. At least 27 health workers were arrested and detained, both inside and outside hospitals [48]. In January 2019, security forces shot and killed a 27-year-old doctor; government security forces entered and raided at least seven hospitals since the protests began; security forces attacked the emergency wards of Omdurman Teaching Hospital and fired tear gas and bullets, resulting in physical injuries among hospital staff [48]. On January 13, 2019, security forces attacked Bahri Teaching Hospital with tear gas and bullets and arrested the administrative director of Al-Faisal Hospital in Khartoum [48].

The Way Out

Economic and educational deterioration, wars, the absence of political leadership, passive media, unemployment, society’s violent tendencies, the poor quality of health services, and the distortion of doctor training have all created a perfect environment for violence against doctors and health facilities. This is happening despite the efforts made by doctors in unhelpful working conditions. The issue is complex and there is no magical solution. Rather, the implementation of a series of reforms and remedies will lead to a reduction in the rate of violence against HCPs. Below, we highlight the solutions suggested in the literature and provide views specific to Sudan.

Solutions to workplace violence against HCPs

Statutory Approach

The WHO recommends that workplace violence prevention should be realized by means of primary, secondary, and tertiary prevention [12]. Statutory approaches toward solutions started in April 1994 when the UK Department of Health allowed the abrupt removal of a patient from a GP’s patient list following an act of actual or threatened violence [2]. Thereafter, the British Medical Association issued guidance stating that: tolerating abusive or violent behavior invites the perpetrator to repeat his or her actions. Therefore, the prevention of violent and threatening behavior is vital to our professionalism [2]. An executive director of health bodies was then nominated to tackle violence against staff and ensure robust training programs, together with the establishment of the Legal Protection Unit and the national incident reporting system, to work with the police, health bodies, and the Crown Prosecution Service to increase the prosecution and conviction of individuals who assault NHS staff [15]. Later, the "zero-tolerance policy" was launched which comprised of statements and warning signs stating that violence would not be tolerated [20]. It communicates explicit boundaries of acceptable behavior and warnings if they are breached, followed by resorting to coercive enforcement, and possible exclusion from services or judicial sanction, if the transgression is serious or repeated [28]. However, it has faced criticism on the grounds of patient stigmatization and breaching of confidentiality, in addition to interference with the doctor-patient relationship caused by measures such as security guards or barricade installation [20,28]. The "Nonviolent Crisis Intervention" was designed to teach HCPs how to prevent and control disruptive behavior. The "Handle with Care" is a collection of self-defense skills and restraining methods for HCPs in potentially assaultive environments [49]. However, a later USA review suggests that stricter penalties be in place for perpetrators and that simple procedures should be implemented to report incidents [6]. In Spain, April 20 is the "National Day Against Aggression in Healthcare Facilities" and the government issued a law to penalize attacks against doctors as a criminal offense of undermining the authority of a government agent [50]. However, criticism of this law is that it allows for the counteraction of patients by filing a parallel complaint for criminal negligence enabling them to reach a final compromise [10]. Similar compromising settlements have been observed post-aggression in Istanbul [15]. In Sudan, it is necessary to implement legislation that facilitates complaint procedures for everyone who believes that a medical error has occurred and to ease follow-up procedures. Furthermore, the Sudanese Medical Council must activate accountability law for healthcare professionals and publish an annual report containing cases of medical errors and the decisions taken. The laws that protect HCPs must be activated and put into effect. Presumably, doctors and nurses tolerate abuse and insults because going to reporting centers causes more suffering and is a waste of time. The inefficiency of the administrative complaint mechanisms in hospitals was an additional factor in under-reporting. It is worth noting that the researchers did not find official statistics in any government hospital for cases of aggression against HCPs, which reflects a relaxed attitude toward this issue. After unprecedented, repeated cases of violence against doctors in Sudan, doctors agreed to carry out a general strike if the law protecting doctors and health personnel was not passed, forcing the transitional government to pass the law in May 2020 [51].

Organizational Procedures

The advantage of introducing organizational improvements to the healthcare system over statutory laws is the fact that they contribute more to the prevention of aggression against HCPs. Preventive action should initially target high-risk HCP groups including the possibility of demographic changes in the physician population, their training on de-escalation techniques, optimizing the patient contact setting, and urging their reporting of every case of aggression [6]. Reduction of lengthy waiting times should be conducted by auditing the appointment system, together with restricting the number of co-patients [2]. Security measures such as unconcealed closed-circuit television with 24-hour video recording and the presence of security staff can help protect hospital property [2]. The emergency departments in public hospitals need a comprehensive development plan and a robust system that accelerates the provision of services within standard time frames thus reducing the unacceptable accumulation of patients within them. During this time frame, the patient should either be treated and discharged, hospitalized, or transferred to another facility. One of the strategies to facilitate patient flow in the ER is to establish a triage. The goal of triage is to risk stratify patient presentations and prioritize the care provided accordingly [52]. Triage helps with the allocation of available resources, such as staff and physical space based on a patient’s clinical needs [52]. Additionally, it is important to establish a computerized system in hospitals allowing for the creation of electronic medical files for each citizen which can be accessed from any medical facility by using a national number. This will speed up patient registration procedures and identify the reasons for previous hospital visits. Likewise, the health system should be connected electronically so that the number of vacant beds in different hospitals can be identified before transferring patients. Currently, there is an urgent need to activate security units in emergency departments.

Increased Expenditure on the Healthcare System

After the success of the Sudan revolution in 2019, there has been a tendency toward reform of the healthcare system. The pressure is toward increasing spending and changing the style of practice. Our fundamental argument is that increased spending on health to an acceptable level will improve first the health of the population and second the morale of citizens, which will be reflected in their behavior.

Responsibility of the Media

HCPs are portrayed negatively by the media, with provocative releases of deaths and sting operations against doctors. It is the media’s responsibility to report to the public that diagnosis is a hypothetical-deductive process, with the need for ongoing questioning and refining, and that there is always a risk of negative outcomes. Doctors cannot be held accountable for every death that occurs in hospitals on account of negligence [10,22].

Responsibility of HCPs

In Sudan, more attention must be given to doctors training in areas like communication skills starting from medical school, internship, and during specialization. We may need to re-consider teaching subjects such as medical ethics and the art of communication with mother tongue language parallel to English. This is consistent with the Mu'tah Declaration [53].

Finally, our review suggests that workplace aggression/violence against HCPs erupts because of interacting factors encompassing staff behavior, patient behavior, and hospital organizational factors. It increases parallel to the increased prevalence of violence in society. To circumvent the problem, a statutory approach and workplace organizational procedures need to be accompanied by robust efforts from HCPs, the media, and other stakeholders. The results of the review describe the findings of previous investigators regarding the definitions, causes, incidents, and solutions to the phenomenon of workplace violence toward HCPs. Although it is difficult to extrapolate from the international experience to fit the situation in Sudan, considering conceptual differences in the interpretation of what constitutes "aggressive behavior" or the understanding of the triggering factors underlying the act, an existing common baseline consensus shall be attempted. First, despite the widespread aggression toward HCPs in Sudan, what motivated this study was the violent life-threatening, and life-taking incidents that became commonplace in the country. Second, the phenomenon is not only problematic for HCPs’ professionalism [2] but is driving them out of the public sector. In fact, one of the contributing factors to the mass immigration of Sudanese doctors [44] is the search for acceptable work environments. The principal demand of the famous 2010 registrar's strike was to improve work environments [54]. The context is comparable to that in India [10,22,23] where expenditure on the healthcare system is meager, subjecting doctors to the reactions of dissatisfied patients. Consistent with previous reports [2,7,21], the wars and political instability witnessed by the country have contributed to this dysfunction, together with associated attributes such as ethnic discordance [31,40]. Presently, the pressure on the government has yielded the issuing and passing of laws protecting HCPs against escalating incidents of workplace violence [51]. This statutory legislative approach was implemented with success in Britain [15,28], the USA [5], and Spain [50].

## Conclusions

The status of workplace violence against HCPs in Sudan is too painful to ignore. It arises from and contributes to, the deterioration of an already challenged public health sector. A multi-approach intervention needs to be integrated to circumvent the standing multi-factorial detrimental situation. Statutory actions are needed toward the widespread violence in the community particularly by unrestrained military personnel who act with impunity and also, toward increasing healthcare expenditure. Workplace organizational procedures are needed to address the patient’s needs that overwhelm scarce resources. Robust educational efforts are recommended by HCP training bodies, the media, and other stakeholders to improve the doctor/patient relationship.
